# Anesthetic management of an infant with arterial tortuosity syndrome undergoing combined laparoscopic procedures: a case report

**DOI:** 10.3389/fped.2026.1929151

**Published:** 2026-10-28

**Authors:** Zainab Abdulaziz Alzayer

**Affiliations:** Department of Anesthesiology, King Fahd University Hospital, Imam Abdulrahman Bin Faisal University, Al Khobar, Saudi Arabia

**Keywords:** arterial tortuosity syndrome, difficult airway, hemodynamic management, infant anesthesia, laparoscopic surgery, case report

## Abstract

**Background:**

Arterial tortuosity syndrome (ATS) is a rare autosomal recessive connective-tissue disorder marked by elongation and tortuosity of major arteries, predisposing affected patients to aneurysm formation, stenosis, and arterial dissection. These vascular abnormalities, together with characteristic craniofacial and connective-tissue features, pose significant anesthetic challenges, particularly in airway management, hemodynamic control, and perioperative cardiovascular risk. The complexity is further heightened in infants undergoing prolonged laparoscopic procedures, in which pneumoperitoneum, reverse-Trendelenburg positioning, and the limited physiological reserve of early infancy may adversely affect ventilation, venous return, and cardiovascular stability.

**Case presentation:**

We describe the anesthetic management of a 3-month-old, 4.6-kg infant with genetically confirmed ATS, who underwent combined laparoscopic repair of a Morgagni diaphragmatic hernia and a para-esophageal hiatal hernia. Preoperative evaluation demonstrated tortuosity of the aorta, head and neck vessels, and pulmonary arteries, without pulmonary hypertension or ventricular dysfunction. Inhalational induction with sevoflurane was performed while preserving spontaneous ventilation because of the anticipated difficult airway and the presence of a Morgagni diaphragmatic hernia, where positive-pressure ventilation could result in gastric insufflation and further distension of herniated abdominal contents. Tracheal intubation was successfully achieved with a 3.0-mm cuffed endotracheal tube. Anesthesia was maintained with sevoflurane and opioid supplementation. Carbon dioxide pneumoperitoneum was introduced gradually, with intra-abdominal pressure maintained below 10 mmHg to limit hemodynamic and ventilatory disturbances. The intraoperative course remained stable, and the patient was extubated uneventfully before transfer to the pediatric intensive care unit.

**Conclusion:**

This case underscores essential anesthetic considerations for infants with ATS undergoing prolonged laparoscopic procedures, emphasizing anticipatory airway planning, vascular-protective strategies, controlled pneumoperitoneum, vigilant cardiovascular management, and coordinated multidisciplinary care.

## Introduction

Arterial tortuosity syndrome (ATS) is a rare autosomal recessive connective tissue disorder caused by pathogenic variants in SLC2A10, which encodes the glucose transporter GLUT10. Loss of functional GLUT10 is believed to impair elastin and collagen maturation in the arterial wall, leading to reduced vascular integrity and marked arterial tortuosity. The condition is characterized by marked elongation and tortuosity of large- and medium-sized arteries, often accompanied by focal stenosis, aneurysm formation, and an increased risk of arterial dissection. Beyond vascular involvement, ATS is frequently associated with a distinctive pattern of connective tissue manifestations, including diaphragmatic hernia, hyperextensible skin, joint laxity, arachnodactyly, and characteristic craniofacial features such as micrognathia and a high-arched palate (1, 3). These multisystem findings have important implications for perioperative management.

Cardiovascular abnormalities are the leading cause of morbidity and mortality in ATS, as diffuse arterial tortuosity and stenotic segments may compromise systemic and pulmonary circulation (1, 3, 5). In addition, craniofacial abnormalities and connective tissue laxity may increase the likelihood of difficult airway management, while vascular fragility raises concern for hemodynamic instability during anesthesia (1, 4, 8). These challenges are further magnified in infants undergoing prolonged laparoscopic procedures, where carbon dioxide pneumoperitoneum and surgical positioning can significantly alter cardiopulmonary physiology.

Published literature on anesthetic management in ATS is extremely limited. To date, only isolated anesthesia-related reports exist, primarily involving older pediatric patients undergoing neurosurgical procedures (4). There are no detailed descriptions of anesthetic management for prolonged laparoscopic surgery in early infancy, despite the unique physiologic demands of this age group.

This report highlights key anesthetic considerations and perioperative management of ATS, with particular emphasis on airway management, hemodynamic stability, and the physiologic effects of prolonged laparoscopy in early infancy.

## Case description

A 3-month-old female infant weighing 4.6 kg was referred for laparoscopic repair of a Morgagni diaphragmatic hernia and a para-esophageal hiatal hernia. She was born at term after an uncomplicated pregnancy and spontaneous vaginal delivery. At presentation, ATS was suspected based on craniofacial features and family history, and the diagnosis was later confirmed by genetic testing. She presented with feeding intolerance and maternal concern about abnormal chest sounds, with no history of cyanosis, tachypnea, aspiration, recurrent respiratory infections, or fever.

The family history was notable for first-degree parental consanguinity and multiple relatives with congenital diaphragmatic hernia, paraesophageal hernia, and tortuous pulmonary arteries. One affected relative, who had no known cardiovascular symptoms before surgery, developed severe postoperative cardiopulmonary deterioration after paraesophageal hernia repair and was subsequently found to have severe pulmonary hypertension and vascular features suggestive of arterial tortuosity syndrome. The clustering of diaphragmatic and paraesophageal hernias, together with vascular abnormalities among affected relatives, further supported the clinical suspicion of ATS before genetic confirmation.

Physical examination revealed several craniofacial and connective tissue features suggestive of ATS, including an elongated facial appearance, micrognathia, a high-arched palate, arachnodactyly, and hyperextensible skin. Chest auscultation revealed bowel sounds over the left hemithorax, and the abdomen was scaphoid, soft, and lax, without organomegaly. Chest radiography showed an air-filled retrocardiac opacity extending into the left lower hemithorax (Figure 1). Contrast-enhanced computed tomography of the chest and abdomen confirmed a para-esophageal hiatal hernia containing stomach and bowel loops within the mediastinum, along with an anterior diaphragmatic defect consistent with a Morgagni hernia (Figure 2). Marked elongation and tortuosity of the aorta and major abdominal vessels were noted, without evidence of aneurysmal dilation.

**Figure 1:**
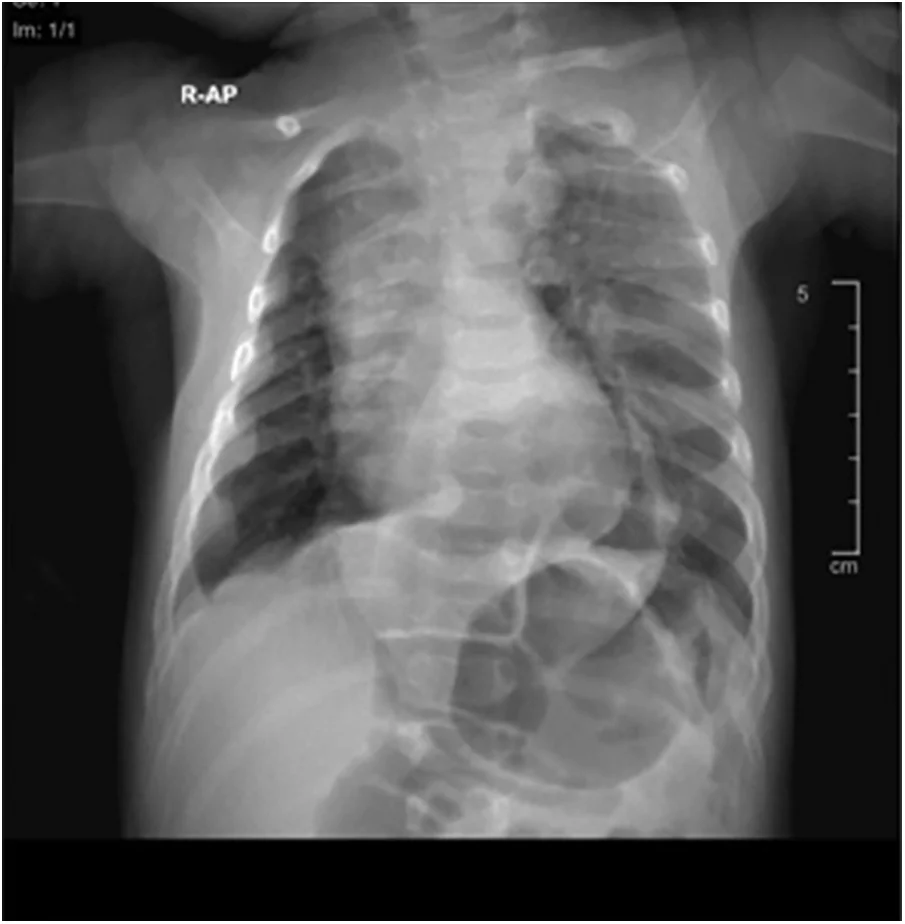
Preoperative chest radiograph demonstrating bowel-containing abdominal viscera within the thorax, consistent with congenital diaphragmatic hernia.

**Figure 2:**
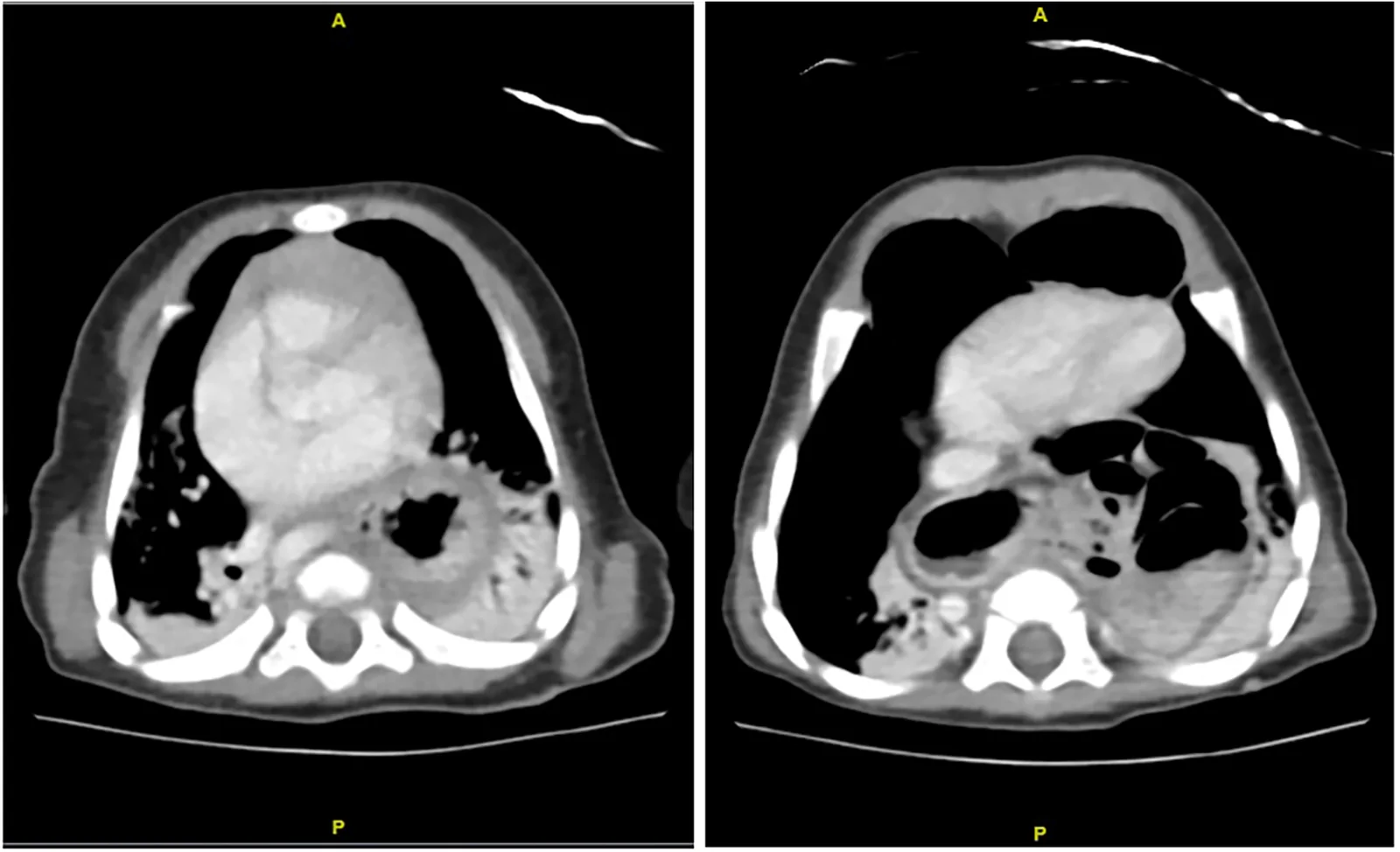
Contrast-enhanced computed tomography demonstrating a paraesophageal hiatal hernia and a Morgagni diaphragmatic hernia with herniation of abdominal contents into the thorax.

Given the vascular findings and family history, a detailed cardiovascular evaluation was performed. Pediatric cardiology consultation and transthoracic echocardiography demonstrated tortuosity of the aortic arch, head and neck vessels, and branch pulmonary arteries, with no pulmonary hypertension, normal biventricular function, and a normal aortic root. These findings informed perioperative planning, which prioritized hemodynamic stability and vascular-protective strategies.

Preoperative fasting followed standard pediatric guidelines. Maintenance fluids (5% dextrose in half-normal saline) were initiated according to standard pediatric maintenance requirements. Active warming measures were implemented due to the infant's low body weight and the anticipated duration of laparoscopy. These measures included forced-air warming, warmed intravenous fluids, an operating room temperature of 22 °C, and continuous core temperature monitoring. Perioperative blood glucose monitoring was also performed.

Standard American Society of Anesthesiologists (ASA) monitoring was applied. Inhalational induction with sevoflurane 6% in 100% oxygen was performed while maintaining spontaneous ventilation because of the anticipated difficult airway and the presence of a Morgagni diaphragmatic hernia. A fully equipped difficult-airway cart was prepared.

After achieving adequate anesthetic depth, a gentle jaw thrust was performed to confirm sufficient depth of anesthesia, and 4 mg of lidocaine was administered to blunt the laryngoscope response. Direct laryngoscopy was performed by the pediatric anesthesia consultant using a Miller size 1 blade, yielding a Cormack–Lehane grade II view and successful placement of a 3.0-mm cuffed endotracheal tube. After intubation, 10 μg of fentanyl and 3 mg of rocuronium were administered, with an additional 5 μg of fentanyl given two hours later. Intravenous paracetamol 65 mg was administered near the end of the procedure as part of a multimodal analgesic regimen.

Hemodynamic stability was maintained throughout induction and airway manipulation. A second ultrasound-guided 24-gauge peripheral intravenous cannula was inserted after intubation to ensure reliable vascular access throughout the procedure. A Foley catheter was inserted for continuous urine output monitoring, and a nasogastric tube was placed for gastric decompression before pneumoperitoneum.

Anesthesia was maintained with sevoflurane delivered in an oxygen–air mixture. Mechanical ventilation was provided in pressure-controlled ventilation with volume guarantee (PCV-VG) mode, with a respiratory rate of 30 breaths/min and positive end-expiratory pressure of 4 cm H₂O. Ventilatory parameters were adjusted to maintain adequate gas exchange during pneumoperitoneum, without significant increases in airway pressures or end-tidal carbon dioxide.

Carbon dioxide pneumoperitoneum was established gradually, with intra-abdominal pressure kept below 10 mmHg to minimize cardiopulmonary perturbations. A total of 40 mL of Ringer's lactate was administered to support preload during pneumoperitoneum and reverse-Trendelenburg positioning. No significant hemodynamic fluctuations were observed during insufflation or positioning.

Estimated blood loss was minimal, and urine output totaled 30 mL. No intraoperative hemodynamic instability, arrhythmias, hypoxemia, or ventilation difficulties occurred. The patient was extubated uneventfully in the operating room and transferred to the pediatric intensive care unit in stable condition.

On postoperative day 1, the patient developed mild atelectasis, managed conservatively with chest physiotherapy. The nasogastric tube was removed on postoperative day 3, and oral feeding was resumed. Recurrent hypoglycemic episodes prompted evaluation by the pediatric endocrinology team, who attributed them to stress-related postoperative hyperinsulinism. Adjusting the feeding regimen led to improvement, and octreotide therapy was initiated.

Follow-up in the genetics and endocrinology clinics showed satisfactory recovery and appropriate weight gain. Whole-exome sequencing performed after surgery confirmed a homozygous pathogenic SLC2A10 variant, establishing the diagnosis of autosomal recessive arterial tortuosity syndrome. The key clinical and perioperative events are summarized in Table 1.

**Table 1:** Timeline of the clinical and perioperative course.

Time point	Key event
Preoperative assessment	ATS suspected based on clinical features and family history. Imaging confirmed Morgagni and paraesophageal hernias. Cardiovascular evaluation demonstrated arterial tortuosity, with no pulmonary hypertension or ventricular dysfunction.
Anesthetic management	Anticipated difficult airway. Inhalational induction with preservation of spontaneous ventilation and successful tracheal intubation.
Intraoperative course	Controlled pneumoperitoneum with stable hemodynamic and respiratory parameters throughout surgery.
Immediate postoperative period	Uneventful extubation and transfer to the PICU in stable condition.
Postoperative course	Mild atelectasis and recurrent hypoglycemia were identified and managed.
Follow-up	Satisfactory recovery. Genetic testing confirmed a pathogenic SLC2A10 variant and the diagnosis of ATS.

## Discussion

In this case, the combination of early infancy, diffuse vascular tortuosity, and the physiologic demands of laparoscopic surgery required a highly individualized anesthetic strategy. Cardiovascular involvement is the principal determinant of perioperative risk in ATS and the major source of morbidity and mortality (1, 3, 5). Diffuse arterial tortuosity and stenotic segments may impair systemic and pulmonary blood flow, increase susceptibility to ischemic events, and predispose patients to arterial dissection (1–3). Although our patient demonstrated tortuosity of the aortic arch, head and neck vessels, and pulmonary arteries without pulmonary hypertension or ventricular dysfunction, the presence of fragile, elongated vessels necessitated meticulous hemodynamic control to avoid abrupt pressure fluctuations. Invasive arterial blood pressure monitoring was considered; however, given the absence of cardiac symptoms, normal ventricular function on echocardiography, and the relatively less invasive nature of the procedure, noninvasive blood pressure monitoring was deemed sufficient.

In addition to the vascular manifestations of ATS, our patient presented with the uncommon coexistence of a Morgagni diaphragmatic hernia and a paraesophageal hiatal hernia. Although diaphragmatic and hiatal hernias have been reported in patients with ATS, the simultaneous occurrence of both defects is rare and further complicates perioperative management (3, 6, 10).

Airway management is another major concern in ATS because of characteristic craniofacial features, including micrognathia, a high-arched palate, and midface retrusion (1, 4, 6, 8). These abnormalities may impair glottic visualization and increase the risk of difficult intubation. Additional airway issues—such as laryngomalacia, subglottic narrowing, and vascular airway compression—have also been described (1). In our patient, several of these features were present, warranting careful airway planning and management by a pediatric anesthesia consultant. The coexistence of a Morgagni diaphragmatic hernia and a para-esophageal hiatal hernia further influenced airway management, as positive-pressure ventilation could result in gastric insufflation and distension of herniated abdominal contents. Therefore, spontaneous ventilation was maintained during inhalational induction until the airway was secured, preserving spontaneous respiratory effort in the event of a difficult airway while minimizing positive-pressure ventilation before tracheal intubation. Although para-esophageal hernias may be associated with gastroesophageal reflux and aspiration, our patient had no history of choking episodes, recurrent aspiration, or recurrent respiratory infections.

Laparoscopic surgery poses additional physiologic challenges in infants with ATS. Carbon dioxide pneumoperitoneum increases intra-abdominal pressure, reduces venous return, elevates systemic vascular resistance, and may impair ventilation (7, 9). These effects are particularly relevant in ATS, where reduced arterial compliance or occult stenotic segments may limit tolerance to hemodynamic fluctuations. Consistent with this concern, pneumoperitoneum in our case was introduced gradually, and intra-abdominal pressure was kept below 10 mmHg to minimize cardiopulmonary disturbance. Prolonged laparoscopy in infancy also increases the risk of hypothermia and hypoglycemia because of limited physiologic reserve; therefore, active warming, glucose monitoring, and careful fluid management were integral to intraoperative care.

Postoperative monitoring is critical in ATS because delayed respiratory or cardiovascular complications can occur, particularly after prolonged anesthesia. In our patient, postoperative intensive-care observation enabled early detection and conservative management of mild atelectasis and recurrent hypoglycemia. The latter required endocrinology consultation and adjustment of feeding strategies, underscoring the importance of multidisciplinary postoperative care.

Only one previous anesthetic case report involving a patient with ATS was identified in the literature. That report described a 13-year-old child undergoing cerebral revascularization surgery, with concerns about airway management and vascular insufficiency. In contrast, our patient was a 3-month-old infant undergoing a prolonged combined laparoscopic repair of concomitant Morgagni and paraesophageal hernias, in which pneumoperitoneum and prolonged operative duration introduced additional anesthetic challenges. Despite differences in age and surgical procedure, both cases underscore the importance of thorough cardiovascular assessment, careful airway planning, and meticulous hemodynamic management in patients with ATS (4). ATS is increasingly recognized in regions with a high prevalence of consanguinity, including the Middle East, where diaphragmatic hernias and diffuse vascular tortuosity have been reported as associated manifestations (6, 10).

This report has limitations inherent to its single-case design. The favorable perioperative outcome observed in this patient cannot establish causality or demonstrate that the anesthetic strategy used is superior to alternative approaches. Furthermore, findings from a single infant cannot be generalized to all patients with ATS because of the heterogeneity in cardiovascular involvement, associated anomalies, and surgical procedures. Therefore, the management described should be considered an individualized approach rather than a universal recommendation.

## Conclusion

This case demonstrates that safe anesthetic management of infants with ATS undergoing prolonged laparoscopic procedures is achievable through comprehensive preoperative cardiovascular assessment, anticipatory airway planning, controlled pneumoperitoneum, and meticulous hemodynamic management. Recognition of characteristic clinical features and suggestive family history can facilitate early suspicion of ATS and prompt evaluation for associated cardiovascular and systemic manifestations. Close postoperative monitoring and coordinated multidisciplinary care further support favorable outcomes in this high-risk population.

## Patient consent

Written informed consent was obtained from the patient's legal guardian for publication of this case report and any accompanying images. A copy of the written consent is available for review by the Editor upon request.

## Data Availability

The original contributions presented in the study are included in the article/Supplementary Material, further inquiries can be directed to the corresponding author.
